# City or Suburb, Resistance Flows: Wastewater-Borne *ESKAPE* and AMR Genes in Malaysian Hospitals

**DOI:** 10.3390/antibiotics14111058

**Published:** 2025-10-23

**Authors:** Sophia Karen Bakon, Nur Fatihah Sholehah Zakaria, Mohd Azerulazree Jamilan, Hazimah Hashim, Zuraifah Asrah Mohamad

**Affiliations:** 1 Health Risk Assessment Unit, Environmental Health Research Centre, Institute for Medical Research, National Institutes of Health, Shah Alam 40170, Selangor, Malaysia; 2 Nutrition Unit, Nutrition, Metabolic and Cardiovascular Research Centre, Institute for Medical Research, National Institutes of Health, Shah Alam 40170, Selangor, Malaysia; 3 Pharmacy Practice and Development Division, Ministry of Health Malaysia, Petaling Jaya 46200, Selangor, Malaysia

**Keywords:** wastewater, effluent, antimicrobial, AMR, hospital, healthcare, resistance genes, resistance bacteria, ESKAPE, residues

## Abstract

Background/Objectives: Hospital wastewater has become a major hotspot for the spread of antimicrobial resistance and the accumulation of antibiotic-resistant genes. We focused on ESKAPE pathogens isolated from hospital wastewater and their antibiotic profiles, which are known to cause nosocomial infections in hospitals. Screening for antibiotic resistant genes in isolates and quantifying antibiotic residues in hospital wastewaters, which may reflect the amount of usage of antibiotics in Klang Valley city hospitals in Malaysia. Methods: Hospital wastewater treatment plants from Klang Valley city were selected based on the study criteria. ESKAPE pathogens were isolated and identified using 16S rRNA PCR, and antibiotic sensitivity testing was performed. Antibiotic resistant gene screenings were performed using quantitative PCR, and quantification of antibiotic residues in the effluent samples was performed using liquid chromatography-mass spectrometry (LC-MS). Results: *Klebsiella pneumoniae* with Multiple Antibiotics Resistance Index (MARi) ranging from low (0.29) to very high resistance (0.71) dominantly isolated among ESKAPE pathogens followed by *Enterococcus faecium* ranging from low (0.29) to critical resistance (1.0) from hospital wastewater in Klang Valley city. The *ermB* gene was the predominant antibiotic resistance gene identified in Klang Valley city hospitals and suburban hospitals, representing 45.5% of isolated *E. faecium* from suburban hospitals and 69% from city hospitals. Although the detection of antibiotic residues was minimal, vancomycin and ciprofloxacin were detected from the wastewater. Conclusions: These findings call for improved wastewater management and antibiotic stewardship to mitigate the spread of resistant pathogens from healthcare facilities into the environment.

## 1. Introduction

Antimicrobial resistance (AMR) is a growing global health crisis, threatening the effectiveness of modern medicine and increasing the burden of infectious diseases. An estimated 4.71 million (95% UI 4.23–5.19) deaths were linked to bacterial antimicrobial resistance (AMR), with 1.14 million (1.00–1.28) deaths directly attributed to bacterial AMR [1]. The transmission risk of β-lactam resistance in nosocomial *Escherichia coli*, particularly the rising resistance to third and fourth generation cephalosporins and carbapenems, has emerged as an important global concern [2,3]. Statistical modelling indicates that among Gram-negative bacteria, resistance to carbapenems has escalated more than any other antibiotic class, increasing from 619,000 associated deaths in 1990 to 1.03 million associated deaths in 2021, and from 127,000 attributable deaths in 1990 to 216,000 attributable deaths in 2021 [1]. Hospitals are critical hotspots for the emergence and dissemination of antimicrobial resistant bacteria (ARB) and antimicrobial resistance genes (ARGs) due to the extensive use of antibiotics for patient treatment [4,5]. Hospital-acquired infections (HAIs), often caused by multidrug-resistant pathogens, further complicate this issue, leading to prolonged hospital stays, increased mortality, and higher healthcare costs [6,7]. Among these, the ESKAPE pathogens (*Enterococcus faecium*, *Staphylococcus aureus*, *Klebsiella pneumoniae*, *Acinetobacter baumannii*, *Pseudomonas aeruginosa*, and *Enterobacter* spp.) are particularly concerning due to their ability to evade conventional treatments and their high prevalence in hospital environments [8,9].

Hospital wastewater, a complex matrix containing pharmaceutical residues, pathogenic microorganisms, and resistant bacterial strains, serves as a significant but often underrecognized contributor to AMR dissemination [10]. Unlike municipal wastewater, hospital effluents contain higher concentrations of antibiotics and multidrug-resistant (MDR) organisms, creating an environment that promotes bacterial adaptation, resistance evolution, and horizontal gene transfer [11]. In developing countries, fast development and population growth may degrade the quality of natural waterbodies as drinking water sources and recipients for wastewater produced by human activities due to the accumulation of bacteria and antibiotic residues [12,13]. Inadequate treatment or direct discharge of hospital wastewater into municipal sewage systems or natural water bodies can facilitate the persistence and spread of resistance determinants, posing serious risks to public health and the environment [14,15].

Antibiotic residues originating from hospitals can persist through conventional treatment processes and enter natural environments [16,17]. These residues exert a selective pressure on microbial communities and become a key driver in the development and propagation of antibiotic resistance [18,19]. The role of hospital wastewater in the environmental dissemination of AMR and HAIs highlights the urgent need for robust monitoring strategies, advanced treatment technologies, and comprehensive regulatory frameworks [20,21]. A study of a high resolution water quality model was performed to assess if the status of technology and wastewater management may halve the proportion of untreated wastewater released to the environment that is adequate by the year 2030 reported that efforts in many regions in the world are still insufficient to achieve the Sustainable Development Goal (SDG) target 6.3 [22,23].

While antibiotic resistance among patients within Malaysian hospitals is being closely monitored, little is known about its relationship with the environment concerning the healthy public. Since there is no conclusive evidence of antibiotic resistance being released from Malaysian hospitals into the environment, a baseline study is very much needed to provide basic knowledge about this loop of transmission. The outcome from this study will fill the gap of important information for clinical practitioners to know if our current hospital is contributing to the source of the AMR problem in the country.

Therefore, this study aims to investigate the presence and spread of antimicrobial resistance (AMR) in Malaysian hospital wastewater. Specifically, it examines the prevalence of antibiotic-resistant bacteria and resistance genes in wastewater discharged from healthcare facilities, quantifying the antibiotic residues that may cause a potential risk to public health and the environment. In conclusion, this study highlights hospital effluents as a significant source of antimicrobial resistance, with resistant bacteria and resistance genes detected at concerning levels. These findings underscore the urgent need for improved wastewater treatment strategies and stricter regulations to minimize the environmental dissemination of resistant pathogens. While this research provides crucial insights, further studies are necessary to investigate the long-term impacts on public health and to develop effective interventions for reducing AMR transmission from healthcare-associated sources.

## 2. Results

### 2.1. In Situ Physical Parameters of Wastewater Testing

This is a cross-sectional study where samplings were done at one occasion of time. The selection of the hospitals was based on the highest usage of antibiotics in Klang Valley city based on the pharmaceutical records for the past five years prior to the start of the study. One hospital (Hospital E) in the suburban area outside the Klang Valley city was chosen for comparison purposes in this study. The physical properties of the wastewater were tested in situ to determine the conditions that support the growth of the isolated bacteria as shown in Table 1. The pH of the wastewater of all the hospitals ranged from pH 6.9 to pH 7.5 which is close to the optimal pH for the neutrophilic ESKAPE pathogens of concern. Wastewater limit ranges for physical parameters vary depending on industrial and environmental discharge guidelines. Hitherto, no regulatory authority established limits for hospital wastewater; thus, these data will be compared to U.S. EPA Industrial Effluent.

### 2.2. Prevalence and Distribution of Antibiotic-Resistant ESKAPE Bacteria

From the bacteria grown on the presumptive selective media, ESKAPE pathogens were identified and confirmed using 16S ribosomal RNA Polymerase Chain Reaction (16S rRNA PCR) as shown in Table 2. *Klebsiella pneumoniae* (*K. pneumoniae*) were commonly isolated from wastewater of hospitals in Klang Valley, followed by *Enterococcus faecium* (*E. faecium*), *Pseudomonas aeruginosa* (*P. aeruginosa*), and *Enterobacter* spp. Meanwhile, *Enterobacter* spp. was commonly found in Hospital E wastewater, followed by *K. pneumoniae*, *E. faecium*, and *P. aeruginosa*. *Acinetobacter baumannii* (*A. baumannii*) was rarely found in hospital wastewater, and no *Staphylococcus aureus* (*S. aureus*) was detected in any of the hospitals.

**Figure 1:**
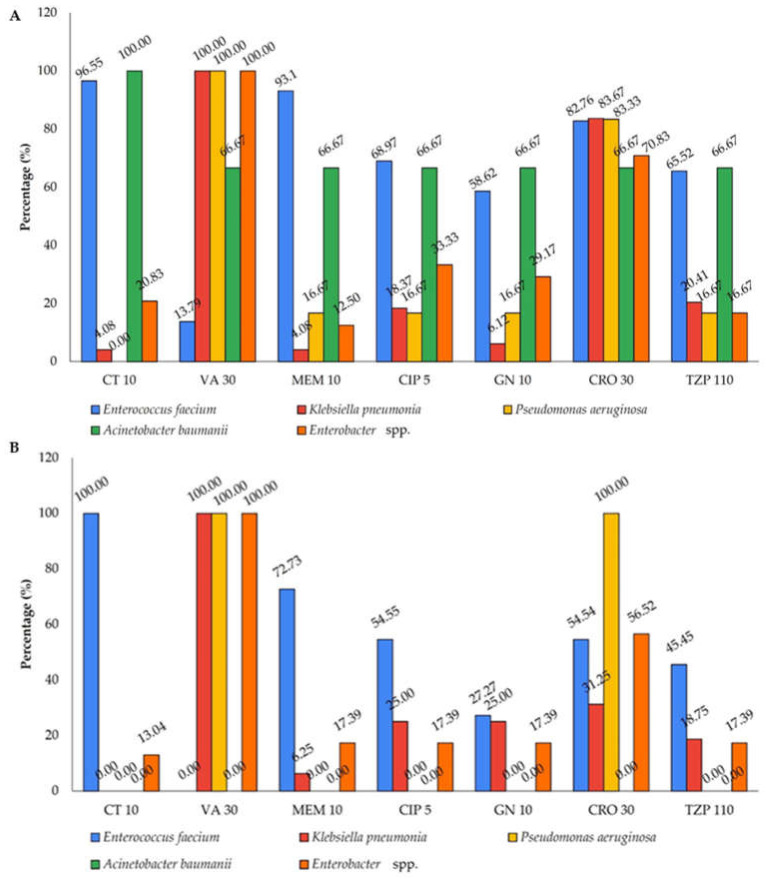
Prevalence of resistant ESKAPE pathogens (**A**) across hospitals in Klang Valley and (**B**) suburban hospital. Resistance was tested against colistin (CT, 10 µg), vancomycin (VA, 30 µg), meropenem (MEM, 10 µg), ciprofloxacin (CIP, 5 µg), gentamicin (GN, 10 µg), ceftriaxone (CRO, 30 µg), and piperacillin-tazobactam (TZP, 110 µg).

The confirmed ESKAPE pathogens were tested against seven types of antibiotics that are widely used in the hospitals. *K. pneumoniae*, *P. aeruginosa*, and *Enterobacter* spp. were 100% resistant to vancomycin (VA 30 µg) and 83% resistant to ceftriaxone (CRO 30 µg) (Figure 1A). It is interesting to note that *E. faecium* has a prevalence of 59% to 96% against six antibiotics, but only 13% against vancomycin (VA 30 µg) across Klang Valley hospitals’ wastewater. In the suburban hospital, *K. pneumoniae*, *P. aeruginosa*, and *Enterobacter* spp. were completely resistant to vancomycin (VA 30 µg), similar to Klang Valley hospitals (Figure 1B). However, *K. pneumoniae* and *Enterobacter* spp. from suburban hospital wastewater had lower prevalence against ceftriaxone by 52% and 14%, respectively, compared to Klang Valley hospitals. The resistance trends of the ESKAPE pathogens against the seven antibiotics were similar between Klang Valley city hospitals and the suburban hospital, although the prevalence rates were lower in the suburban hospital for most of the pathogen except for *E. faecium* against colistin and *P. aeruginosa* against ceftriaxone.

### 2.3. Antibiotic Susceptibility Profiles and Multidrug Resistance Trends

**Figure 2:**
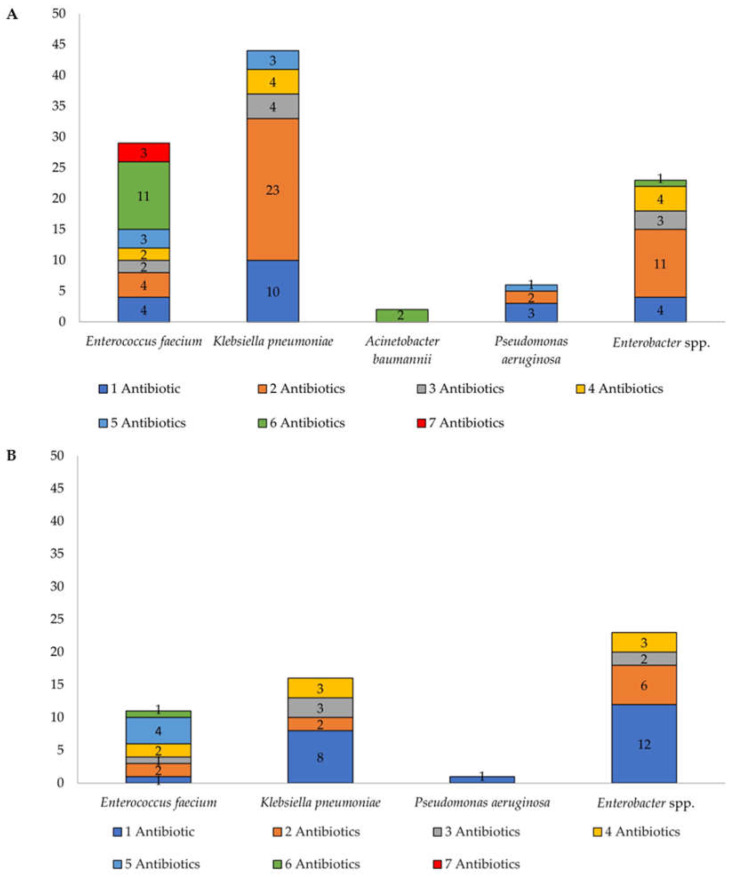
Number of ESKAPE pathogens resistant to 7 types of antibiotics isolated from (**A**) Klang Valley city hospitals’ wastewater and (**B**) suburban hospital.

Overall, ESKAPE pathogens isolated from Klang Valley city hospitals’ wastewater demonstrated resistance to one to six antibiotics, with most of the pathogens resistant to two antibiotics (Figure 2A). *E. faecium* had many isolates resistant to six antibiotics, and three of them were completely resistant to all seven antibiotics tested. In the suburban hospital, *E. faecium* isolates were resistant to one to five types of antibiotics, with most of the isolates resistant to five types of antibiotics and the rest of the pathogens largely resistant to only one type of antibiotic (Figure 2B).

#### Multiple Antibiotic Resistance Index (MARi)

**Figure 3:**
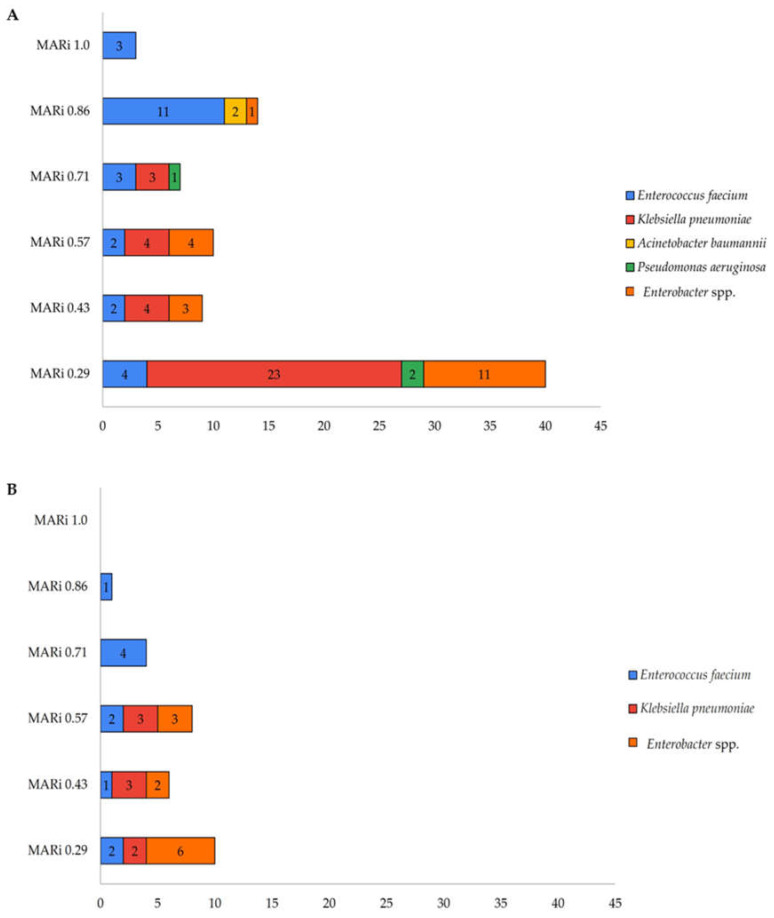
Number of isolates based on MARi of ESKAPE pathogens resistant to two and more antibiotics tested. (**A**) EKAPE pathogens were found to be resistant to at least two types of antibiotics isolated from hospitals’ wastewater in Klang Valley city (**B**) EKE pathogens were resistant to two and more antibiotics isolated from suburban hospital’s wastewater. The MAR-index values are categorized as the following: 0–0.29 (Low), 0.43 (Moderate), 0.57 (High), 0.71 (Very High), and 0.86–1.0 (Critical resistance).

The Multiple Antibiotic Resistance Index (MARi) was calculated for each isolate using the formula MAR = a/b, where a represents the number of antibiotics to which the test isolate demonstrated resistance and b represents the total number of antibiotics to which the test isolate was tested for susceptibility. The MAR-index values for ESKAPE pathogens from the Klang Valley city hospitals’ wastewater (Figure 3A) were *E. faecium* 0.29–1.0, *K. pneumoniae* 0.29–0.71, *A. baumannii* 0.86, *P. aeruginosa* 0.29–0.71, and *Enterobacter* spp. 0.29–0.86. For the suburban hospital wastewater (Figure 3B), the MAR-index values were *E. faecium* 0.29–0.86, *K. pneumoniae* 0.29–0.57, and *Enterobacter* spp. 0.29–0.57. The MAR-index values are categorized as the following: 0–0.29 (Low), 0.43 (Moderate), 0.57 (High), 0.71 (Very High), and 0.86–1.0 (Critical resistance).

### 2.4. Detection and Prevalence of Antibiotic Resistance Genes (ARGs)

All of the isolated ESKAPE pathogens were screened for six antibiotic resistance genes (ARGs), namely, *VanA*, *BlaTEM*, *ermB*, *tetA*, *Sul1*, and *BlaNDM-1*. The highest prevalence of ARG from Klang Valley city hospitals’ wastewater was the *ermB* gene (69.0%), followed by *VanA* (10.3%) and *BlaNDM-1* present in *E. faecium* isolates (Table 3). As for *K. pneumoniae*, the prevalence of ARGs that were present were *BlaTEM* (38.8%), *tetA* (38.8%), *Sul1* (34.7%), *ermB* (18.4%), and *BlaNDM-1* (14.30%). Out of many ARGs screened from *A. baumannii*, only *BlaTEM* was detected from the isolate. The ARGs carried in the *P. aeruginosa* were *BlaTEM* (16.7%), *tetA* (16.7%), *Sul1* (16.7%), and *BlaNDM-1* (33.3%). *Enterobacter* spp. were found to have high prevalence of *Sul1* (58.3%), followed by *ermB* (16.7%), *BlaTEM* (12.5%), *tetA* (8.3%), and *BlaNDM-1* (4.2%).

The *E. faecium* isolates from the suburban hospital’s wastewater likewise had a high presence of *ermB* (46.0%) (Table 4), but at a lower prevalence than the Klang Valley city hospitals’ wastewater, followed by *BlaNDM-1* (9.1%), and no *VanA* was detected in the isolates. In comparison to the isolates from Klang Valley city hospitals’ wastewater, *Sul1* (50.0%) had the highest prevalence of ARGs carried by *K. pneumoniae* followed by *BlaTEM* (43.8%), *tetA* (31.3%), *BlaNDM-1* (18.8%), and *ermB* (12.5%). Out of six ARGs screened, only *Sul1* was detected which was carried by the *P. aeruginosa* isolate from the suburban hospital’s wastewater. The *Sul1* (52.2%) gene was the most frequently detected in *Enterobacter* spp. similarly to isolates from the city hospitals. However, unlike the city hospitals, where *BlaNDM-1* was present and *VanA* was absent, no *BlaNDM-1* was detected, but *VanA* (13%) was present instead.

### 2.5. Quantification and Antibiotic Residues Levels in Hospital Effluents

The study of antibiotic residues was performed according to the established detection range of our developed method. The detection of antibiotics in the collected Hospital’s wastewater samples, including effluent and influent sampling points, was determined according to the concentration range of 1000–5000 µg/L for colistin (CT), 100.0–500.0 µg/L for vancomycin (VA), 0.10–0.50 µg/L for meropenem (MEM), 1.0–5.0 µg/L for ciprofloxacin (CIP), 5–25.0 µg/L for ceftriaxone (CRO), 0.50–2.50 µg/L for tazobactam (TZ), and 0.10–0.50 µg/L for piperacillin (P), and with their respective detection limit (DL) of 2990 µg/L, 61 µg/L, 0.05 µg/L, 0.3 µg/L, 2.1 µg/L, 0.16 µg/L, and 0.02 µg/L.

The summary of antibiotic residues found from several antibiotics was tabulated in Table 5 below. For instance, the antibiotics of CT, MEM, CRO, and P were not found in any of the hospitals’ wastewater samples. In contrast, CIP was found in all Klang Valley city hospitals’ wastewater, but not in Hospital E (suburban). Interestingly, only TZ was detected from the Hospital E wastewater sample. On the other hand, although very minimal, VA was detected only from wastewater in Klang Valley city hospitals, namely, Hospital A, Hospital B, and Hospital C.

## 3. Discussion

The analysis of hospital wastewater effluents across five hospitals revealed that *K. pneumoniae* was the most frequently detected ESKAPE pathogen, particularly dominant in Hospital B (25.77%) and found in all facilities, including the suburban Hospital E. *E. faecium* was the second most common, with the highest prevalence in Hospital C (16.95%) [24,25]. *K. pneumoniae* is known for its remarkable genome plasticity, which enables it to rapidly acquire and integrate foreign genetic material, including antimicrobial ARGs. This plasticity facilitates horizontal gene transfer through plasmids, integrons, and transposons, allowing the bacterium to adapt swiftly to antibiotic pressure [26]. Due to its high genome plasticity, *K. pneumoniae* can survive in wastewater and other contaminated environments, where it acts as a reservoir and amplifier of antimicrobial resistance traits, significantly contributing to the environmental spread of AMR [27]. Our study found that *E. faecium* was second-most prevalent pathogen isolated from the hospitals’ wastewater. *E. faecium* is a nosocomial pathogen frequently linked to bloodstream infections, urinary tract infections, and surgical site infections. Strains isolated from hospitals often demonstrate multidrug resistance, and when these resistant strains are excreted by patients and enter the hospital wastewater system, they transport antimicrobial resistance genes (ARGs) that can endure conventional wastewater treatment processes, as reported in European hospital wastewater [25]. *Enterobacter* spp. also showed significant presence, notably in Hospital D (15.38%) and even higher in the suburban site (15.86%), suggesting widespread environmental dissemination [28]. *P. aeruginosa* was detected at low levels in most hospitals, while *A. baumannii* was found only in Hospital D and *S. aureus* was not detected in any of the wastewater samples. These findings may be due to the various range of temperature and media conditions to grow the mentioned bacteria that are not easily isolated from the environment compared to the clinical strains [29]. Considering the standardized temperature protocol we set during the incubation, which is 37 °C, it may be causing the limitation for these two bacteria to be isolated due to the original temperature of the isolates being in the range of 26 °C to 30 °C. The findings demonstrate varied yet concerning levels of ESKAPE pathogens in hospital effluents from both urban and suburban settings, with specific species showing localized concentration hotspots. Supporting this, a study conducted on hospital wastewater in Mexico found that bacterial genera from the ESKAPEE group were abundant in treated effluents, highlighting their resistance to treatment processes and elevated risk of environmental dissemination due to antibiotic resistance [30].

Across Klang Valley city hospitals, *E. faecium* and *A. baumannii* showed high resistance to colistin (CT), while *K. pneumoniae*, *Enterobacter* spp., and *P. aeruginosa* exhibited 100% resistance to vancomycin (VA). This may be due to *K. pneumoniae* develops colistin resistance through chromosomal mutations example; *mgrB*, *pmrAB* and plasmid-mediated *mcr* genes that alter its outer membrane, reducing drug binding [31,32]. In contrast, *E. faecium* is intrinsically resistant to colistin due to the absence of lipopolysaccharides and an outer membrane, which are essential for colistin’s antibacterial action [33]. Notably, *K. pneumoniae* and *Enterobacter* spp. showed moderate resistance to meropenem (MEM), ciprofloxacin (CIP), and ceftriaxone (CRO), with *E. faecium* showing particularly high resistance to CRO and GN. In the suburban hospital, *E. faecium* again demonstrated complete resistance to CT, while *K. pneumoniae* and *Enterobacter* spp. displayed full resistance to VA and 56% and 31% resistance to CRO, respectively. *K. pneumoniae* and *Enterobacter* spp. resistance to MEM, CIP, GN, and TZP was lower overall in the suburban hospital, except for elevated ceftriaxone resistance in *P. aeruginosa* (100%). These results highlight widespread resistance among key pathogens, with variations in resistance intensity across hospital settings and bacterial species [34,35].

The ESKAPE pathogen isolates from hospital wastewater were categorized by their Multiple Antibiotic Resistance Index (MARi) values. In Klang Valley city hospitals, a broad range of MARi values was observed, with the highest number of isolates falling within the low resistance category (MARi 0.29) dominated by *Klebsiella pneumoniae* (23 isolates) and *Enterobacter* spp. (11 isolates). These isolates have likely been exposed to lower levels of antibiotics in their environment and hospital settings, indicating reduced selective pressure for resistance potentially reflecting effective antibiotic stewardship practices [36,37]. Notably, isolates with MARi 0.86–1.0 were also present, including *E. faecium* (14 isolates) and *A. baumannii* (2 isolates), and one *Enterobacter* spp. appeared in the critical resistance categories. The presence of such high-MAR index isolates suggests prolonged or repeated exposure to multiple classes of antibiotics, possibly due to heavy clinical usage or poor wastewater management. *E. faecium* and *A. baumannii*, both members of the ESKAPE group that cause nosocomial infections, are known for their intrinsic resistance and ability to acquire additional resistance mechanisms, contributing to their persistence in hospital effluents [38].

In contrast, the suburban hospital exhibited fewer overall resistant isolates, with the majority also clustering at low MARi values (0.29 and 0.43). However, *Enterobacter* spp. remained a prominent contributor to resistance at all levels. *E. faecium* showed a small number of isolates but with critical and very high resistance, and *K. pneumoniae* appeared in moderate to high MARi categories maybe due to overexpression of antibiotic resistance genes in hospital effluents over time as reported in a spatiotemporal study within the river catchment to determine the contribution of ARG originating from various sources including hospitals’ effluents [39].

These findings suggest that while both urban and suburban hospitals harbor multidrug-resistant organisms, the Klang Valley hospitals present a broader and more severe spectrum of antibiotic resistance, indicating a higher potential risk of resistant pathogen dissemination [40]. Klang Valley city hospitals typically manage higher patient loads and more complex clinical cases, which often necessitate broad-spectrum or prolonged antibiotic use. Prior to the start of the study, some references from the Pharmacy Practice and Development Division, Ministry of Health Malaysia of the Defined Daily Dosage/1000 patient day (DDD/1000) were consulted, and the hospitals that were selected were exceeding the upper limit for the antibiotics as follows: CT (15.06), VA (12.49), MEM (88.47), CIP (4.73), GN (0.24), CRO (62.04), and TZP (49.16). This increased their consumption and may create stronger selective pressure for resistant bacteria and ARGs to proliferate. The hospitals in Klang Valley city serve densely populated areas, likely resulting in a higher incidence of nosocomial infections. Frequent antibiotic treatments contribute to resistance development and ARG enrichment in wastewater [41]. A comparative analysis of hospital effluents revealed higher diversity and prevalence of antibiotic resistance genes (ARGs) in Klang Valley hospitals than in a suburban hospital. *K. pneumoniae* in Klang Valley exhibited the most ARGs, notably *BlaTEM*, *tetA*, *Sul1*, *ermB*, and *BlaNDM-1*, while *Enterobacter* spp. and *E. faecium* also carried multiple ARGs. In the suburban hospital, *Enterobacter* spp. and *K. pneumoniae* remained dominant ARG carriers, with *Sul1*, *tetA*, and *BlaTEM* frequently detected. *S. aureus* was not detected across all sites, and *A. baumannii* was rarely positive. These findings emphasize the urban and suburban differences in the distribution of antibiotic resistance genes (ARGs), influenced by the extensive use of antibiotics. Hospital wastewater serves as a reservoir for the majority of ARGs and contributes to the diversity of ARGs in the surrounding natural environments as reported in Sweden [42]. Overall, *K. pneumoniae* and *Enterobacter* spp. were the most consistent carriers of multiple ARGs in both urban and suburban hospital settings. However, ARG prevalence was generally higher in the Klang Valley, possibly reflecting increased antimicrobial use or selective pressure in more densely populated healthcare environments. Although the mean detection was lower than the detectable limit, vancomycin and ciprofloxacin residues’ occurrence in Klang Valley city hospitals suggests that the high usage of the antibiotics is to treat nosocomial infection [43,44]. These findings highlight the importance of localized surveillance to inform targeted AMR mitigation strategies.

## 4. Materials and Methods

A cross-sectional study was conducted by collecting wastewater from 4 tertiary hospitals in Klang Valley, Malaysia. The hospitals were selected based on these study criteria: highest usage of antibiotics reported by Pharmacy Practice and Development Division, Ministry of Health Malaysia for the past 5 years before the start of the study and having its individual hospital sewage treatment plant (only treating wastewater from the hospital blocks) and not communal sewage treatment plant (receiving wastewater from surrounding populations nearby) [45]. In situ wastewater physical parameters were tested to understand the nature for the pathogens to survive and multiply in the environment.

### 4.1. Hospital Wastewater Sampling

Four tertiary hospitals in Klang Valley are among the busiest city hospitals in Malaysia and were selected as study sites for wastewater effluent sampling. In this study, we included one hospital outside the Klang Valley for a comparison with the suburban area. A sterile water scoop and a 1 l sterile screw-capped Schott Duran (Mainz, Germany) amber bottle were used to collect a 1 litre effluent wastewater sample for microbiological analysis, and another 1 litre for antimicrobial residue quantification analysis. The hospital wastewater was collected from the effluent discharge point of the sewage treatment plant. During the sampling activities, the physical condition of the wastewater effluent was recorded, and the in situ wastewater physical parameters were tested. The wastewater samples were stored in a cold box at 4 °C to maintain their integrity during the transportation to the laboratory for immediate processing.

### 4.2. Bacteria Isolation and Identification

For each wastewater sample, tenfold serial dilutions were made in 50 mL of sterile saline solution. Ten mL of the sample was filtered through sterile 0.45 µm pore size cellulose acetate filters (Sartorius Singapore Pte Ltd., Singapore) in triplicate following the protocol from Bakon et. al [45]. The filter then was placed onto the selective agar media to isolate ESKAPE pathogens incubated at 37 °C for 24 to 48 h. The identification of the colonies grown on the filter paper was done using the 16S RNA polymerase chain reaction (PCR) technique using a set of universal primers (forward, CCT ACG GGA GGC AGC AG; reverse, ATT ACC GCG GCT GCT GG) under the following conditions: polymerase activation at 94 °C for 2 min, denaturation at 94 °C for 5 s, annealing at 60 °C for 10 s, extension at 72 °C for 20 s, and holding at 4 °C, and the PCR products were sent for sequencing.

### 4.3. Antibiotic Sensitivity Testing

The positively identified ESKAPE colonies were subjected to antimicrobial susceptibility testing (AST) using the disk diffusion method (Kirby–Bauer), following the Clinical Laboratory Standards Institute (CLSI) guidelines. The AST experiments were performed in triplicate for each colony, with a multidrug-resistant isolate included as a technical control in every test. The antibiotics used for AST included ceftriaxone (30 µg), ciprofloxacin (5 µg), meropenem (10 µg), vancomycin (30 µg), colistin (10 µg), and piperacillin/tazobactam (110 µg). For bacteria not listed in the CLSI guidelines, measurements were interpreted according to the European Committee on Antimicrobial Susceptibility Testing (EUCAST) guidelines.

### 4.4. Antibiotic Resistance Gene Screening

ESKAPE pathogens that were isolated were cultured and inoculated in tryptone soy broth medium (CM0129, Oxoid, Basingstoke, UK). The cultures were incubated at 37 °C for 18–24 h. The genomic DNA were extracted using the PureGene Bacteria Kit (Qiagen, Hilden, Germany) following the manufacturer’s instructions. The concentration of the extracted DNA was measured using a Multiskan SkyHigh microplate spectrophotometer (Thermo Scientific, Waltham, MA, USA) before qPCR amplification. The PCR conditions were optimized according to published methods (Supplementary Table S1). The size of the amplified products was confirmed by 3% agarose gel (NextGene, Selangor, Malaysia) electrophoresis at 90 V for 90 min. The PCR products were sent to an outsourcing service for Sanger sequencing. The sequencing results were analyzed using the Basic Local Alignment Search Tool nucleotide (BLASTn) tool in the National Center for Biotechnology Information database to identify the correct amplified relevant genes.

### 4.5. Quantification of Antibiotic Residues

The concentration of antibiotic residues was determined using liquid chromatography-mass spectrometry (LCMS) with an ion source of a Heated Electrospray Ionization (HESI II) probe (Q-Exactive, Thermo Scientific, Waltham, MA, USA). The separation was performed using a C18 analytical column with 150 mm × 2.1 mm dimension (ACE EXCEL 3, Avantor-ACE—Advanced Chromatography Technologies, Radnor, PA, USA) at 0.8 mL/min with a fixed injection volume of 60 µL. The chromatographic condition was set up based on LCMS-grade water and acetonitrile in 0.1% (*v*/*v*) formic acid as mobile phases A and B, respectively. The solvent gradient with a total runtime of 23 min was as follows: the mobile phase B was fixed at 5% for 1 min, then ramped up to 30% until 12 min. Then, it was further increased to 100% for another 1 min. The condition was maintained at 100% to 17 min, followed by an immediate decrease to 5% until 23 min.

## 5. Conclusions

To our knowledge, this work represents the first study to combine the antibiotic resistance profile of ESKAPE pathogens and the detection of antibiotic-resistant genes carried by the pathogens and quantifying antibiotic residues from hospitals’ wastewater in Malaysia. This study provides compelling evidence that hospital wastewater, particularly from urban facilities in the Klang Valley, serves as a significant reservoir and conduit for the dissemination of antimicrobial-resistant ESKAPE pathogens and associated resistance genes. *K. pneumoniae* emerged as the most prevalent and resistant species across all sites, demonstrating high genome plasticity and widespread ARG carriage, especially in urban hospitals. *E. faecium* and *Enterobacter* spp. were also frequently detected, with *E. faecium* exhibiting high resistance levels and *Enterobacter* spp. showing broad environmental distribution. Notably, MAR index analysis revealed both low and critically high resistance profiles, with urban hospitals harboring a greater diversity and intensity of multidrug resistance.

The detection of ARGs such as *blaTEM*, *blaNDM-1*, *tetA*, *sul1*, and *ermB* in dominant species underscores the role of untreated or inadequately treated hospital effluents in propagating AMR into the environment. The elevated antibiotic consumption in Klang Valley hospitals, confirmed by DDD metrics, likely contributes to the higher resistance burden observed. Although vancomycin and ciprofloxacin residues were below detection thresholds, their presence reflects ongoing selective pressure within these clinical settings.

In summary, the findings highlight a clear urban–suburban divide in AMR prevalence and diversity, with urban hospitals posing a greater risk for environmental dissemination of resistant pathogens. This underscores the urgent need for strengthened surveillance, advanced wastewater treatment technologies, and antibiotic stewardship programs, particularly in densely populated healthcare hubs. Localized and sustained interventions are essential to mitigate the environmental spread of AMR and protect public and ecosystem health.

## Figures and Tables

**Table 1:** In situ water quality parameters of hospital wastewater effluents.

Parameter	Hospital A	Hospital B	Hospital C	Hospital D	Hospital E (Suburban)
pH	6.99 ± 0.03	7.46 ± 0.11	7.51 ± 0.04	7.42 ± 0.09	7.14 ± 0.19
Conductivity (µS/cm)	532.67 ± 4.16	373.33 ± 0.58	331.00 ± 0	596.33 ± 0.58	633.33 ± 4.73
Turbidity (NTU)	50.27 ± 1.27	78.63 ± 1.57	8.55 ± 1.01	0.19 ± 0.01	30.07 ± 0.65
Free Chlorine (mg/L)	0.2 ± 0.03	0.05 ± 0.006	0.02 ± 0.02	0.16 ± 0.03	0.02 ± 0.01
Total Chlorine (mg/L)	0.22 ± 0.006	0.07 ± 0.02	0.02 ± 0.01	0.13 ± 0.01	0.04 ± 0.01
Temperature (°C)	27.33 ± 0.61	26.83 ± 0.21	28.60 ± 0.00	25.87 ± 0.64	30.30 ± 0.26

The data are presented as mean ± standard deviation.

**Table 2:** Prevalence of ESKAPE bacteria in hospital wastewater effluents identified using 16S rRNA PCR.

Pathogen	Hospital A	Hospital B	Hospital C	Hospital D	Total (Hospital A–D)	Hospital E (Suburban)
***Enterococcus faecium***	8.97 (7/78)	5.15 (5/97)	16.95 (10/59)	10.77 (7/65)	9.70 (29/299)	7.60 (11/145)
***Staphylococcus aureus***	0	0	0	0	0	0
***Klebsiella pneumoniae***	19.23 (15/78)	25.77 (25/97)	3.39 (2/59)	10.77 (7/65)	16.39 (49/299)	11.03 (16/145)
***Acinetobacter baumannii***	0	0	0	4.62 (3/65)	1.00 (3/299)	0
***Pseudomonas aeruginosa***	1.28 (1/78)	2.06 (2/97)	3.39 (2/59)	1.54 (1/65)	2.00 (6/299)	0.70 (1/145)
***Enterobacter*** **spp.**	3.85 (3/78)	7.22 (7/97)	6.8 (4/59)	15.38 (10/65)	8.03 (24/299)	15.86 (23/145)

Data are presented as prevalence (%) and absolute counts (*n*/N). N = Total of bacteria grown on presumptive selective media.

**Table 3:** Prevalence of ARGs in hospital effluents from Klang Valley hospitals.

Antibiotic Resistant Genes (ARGs)	***Enterococcus faecium***	***Staphylococcus aureus***	***Klebsiella pneumoniae***	***Acinetobacter baumannii***	***Pseudomonas aeruginosa***	***Enterobacter*** spp.
*VanA*	10.3 (3/29)	ND	ND	ND	ND	ND
*BlaTEM*	ND	ND	38.8 (19/49)	33.3 (1/3)	16.7 (1/6)	12.5 (3/24)
*ermB*	69.0 (20/29)	ND	18.4 (9/49)	ND	ND	16.7 (4/24)
*tetA*	ND	ND	38.8 (19/49)	ND	16.7 (1/6)	8.3 (2/24)
*Sul1*	ND	ND	34.7 (17/49)	ND	16.7 (1/6)	58.3 (14/24)
*BlaNDM-1*	3.5 (1/29)	ND	14.3 (7/49)	ND	33.3 (2/6)	4.2 (1/24)

Data are presented as prevalence (%) and absolute counts (*n*/N). Abbreviation: ND, not detected.

**Table 4:** Prevalence of ARGs in hospital effluents from suburban hospital.

Antibiotic Resistant Genes (ARGs)	***Enterococcus faecium***	***Staphylococcus aureus***	***Klebsiella pneumoniae***	***Acinetobacter baumannii***	***Pseudomonas aeruginosa***	***Enterobacter*** spp.
*VanA*	ND	ND	ND	ND	ND	13.0 (3/23)
*BlaTEM*	ND	ND	43.8 (7/16)	ND	ND	8.7 (2/23)
*ermB*	45.5 (5/11)	ND	12.5 (2/16)	ND	ND	13.0 (3/23)
*tetA*	ND	ND	31.3 (5/16)	ND	ND	26.1 (6/23)
*Sul1*	ND	ND	50.0 (8/16)	ND	100 (1/1)	52.2 (12/23)
*BlaNDM-1*	9.1 (1/11)	ND	18.8 (3/16)	ND	ND	ND

**Table 5:** Antibiotic residues detected from hospitals’ wastewater effluent.

Antibiotics							
Sampling Source (Wastewater)	CT (µg/L) (1000–5000 µg/L; R^2^ = 0.9803; DL 2990 µg/L)	VA (µg/L) (100–500 µg/L; R^2^ = 0.9860; DL 61 µg/L)	MEM (µg/L) (0.10–0.50 µg/L; R^2^ = 0.9940; DL 0.05 µg/L)	CIP (µg/L) (1.0–5.0 µg/L; R^2^ = 0.9953; DL 0.3 µg/L)	CRO (µg/L) (5.0–25.0 µg/L; R^2^ = 0.9692; DL 2.1 µg/L)	TZ (µg/L) 0.50–2.50 µg/L; R^2^ = 0.9992; DL 0.16 µg/L)	P (µg/L) (0.10–0.50 µg/L; R^2^ = 0.9917; DL 0.02 µg/L)
Hospital A	ND	0.091 ± 0.128 *	ND	0.019 ± 0.003 *	ND	ND	ND
Hospital B	ND	0.193 ± 0.273 *	ND	0.627 ± 0.003	ND	ND	ND
Hospital C	ND	0 ± 0.520 *	ND	0.041 ± 0.001 *	ND	ND	ND
Hospital D	ND	ND	ND	0.080 ± 0.014 *	ND	ND	ND
Hospital E	ND	ND	ND	ND	ND	0.393 ± 0.005	ND

* The reported mean was below the DL (detection limit).

## Data Availability

The research dataset is available from the corresponding author on reasonable request due to privacy or ethical restrictions.

## Supplementary Materials

The following supporting information can be downloaded at: https://www.mdpi.com/article/10.3390/antibiotics14111058/s1, Table S1: Primer sequence, annealing temperature, Positive control and CT-values.

## Abbreviations

The following abbreviations are used in this manuscript:

AMR	Antimicrobial resistance
ARB	Antimicrobial resistant bacteria
ARGs MDR	Antimicrobial resistant genes Multidrug-resistant
HAIs	Hospital-acquired infections
ESKAPE	*Enterococcus faecium*, *Staphylococcus aureus*, *Klebsiella pneumoniae*, *Acinetobacter baumannii*, *Pseudomonas aeruginosa*, and *Enterobacter* spp.
CLSI	Clinical Laboratory Standards Institute
EUCAST	European Committee of Antimicrobial Susceptibility Testing
PCR	Polymerase Chain Reaction
qPCR	quantitative Polymerase Chain Reaction
DNA	Deoxyribonucleic acid
BLASTn	Basic Local Alignment Seacrh Tool Nucleotide
LCMS	Liquid Chromatography-mass Spectrometry

## References

[1] Naghavi M., Vollset S.E., Ikuta K.S., Swetschinski L.R., Gray A.P., Wool E.E., Robles Aguilar G., Mestrovic T., Smith G., Han C. (2024). Global burden of bacterial antimicrobial resistance 1990–2021: A systematic analysis with forecasts to 2050. Lancet.

[2] Chukwu E.E., Awoderu O.B., Enwuru C.A., Afocha E.E., Lawal R.G., Ahmed R.A., Olanrewaju I., Onwuamah C.K., Audu R.A., Ogunsola F.T. (2022). High prevalence of resistance to third-generation cephalosporins detected among clinical isolates from sentinel healthcare facilities in Lagos, Nigeria. Antimicrob. Resist. Infect. Control.

[3] Gregova G., Kmet V. (2020). Antibiotic resistance and virulence of Escherichia coli strains isolated from animal rendering plant. Sci. Rep..

[4] Hutinel M., Fick J., Larsson D.G.J., Flach C.-F. (2021). Investigating the effects of municipal and hospital wastewaters on horizontal gene transfer. Environ. Pollut..

[5] Levin-Reisman I., Brauner A., Ronin I., Balaban N.Q. (2019). Epistasis between antibiotic tolerance, persistence, and resistance mutations. Proc. Natl. Acad. Sci. USA.

[6] Metersky M.L., Kalil A.C. (2017). New guidelines for nosocomial pneumonia. Curr. Opin. Pulm. Med..

[7] Stewart S., Robertson C., Pan J., Kennedy S., Haahr L., Manoukian S., Mason H., Kavanagh K., Graves N., Dancer S.J. (2021). Impact of healthcare-associated infection on length of stay. J. Hosp. Infect..

[8] Tacconelli E., Carrara E., Savoldi A., Harbarth S., Mendelson M., Monnet D.L., Pulcini C., Kahlmeter G., Kluytmans J., Carmeli Y. (2018). Discovery, research, and development of new antibiotics: The WHO priority list of antibiotic-resistant bacteria and tuberculosis. Lancet Infect. Dis..

[9] Balasubramanian R., Van Boeckel T.P., Carmeli Y., Cosgrove S., Laxminarayan R. (2023). Global incidence in hospital-associated infections resistant to antibiotics: An analysis of point prevalence surveys from 99 countries. PLoS Med..

[10] Kaur R., Yadav B., Tyagi R.D., Tyagi R.D., Sellamuthu B., Tiwari B., Yan S., Drogui P., Zhang X., Pandey A. (2020). 4-Microbiology of hospital wastewater. Current Developments in Biotechnology and Bioengineering.

[11] Petrovich M.L., Zilberman A., Kaplan A., Eliraz G.R., Wang Y., Langenfeld K., Duhaime M., Wigginton K., Poretsky R., Avisar D. (2020). Microbial and Viral Communities and Their Antibiotic Resistance Genes Throughout a Hospital Wastewater Treatment System. Front. Microbiol..

[12] Mohamad Z.A., Bakon S.K., Jamilan M.A.J., Daud N., Ciric L., Ahmad N., Muhamad N.A. (2022). Prevalence of Antibiotic-Resistant Bacteria and Antibiotic-Resistant Genes and the Quantification of Antibiotics in Drinking Water Treatment Plants of Malaysia: Protocol for a Cross-sectional Study. JMIR Res. Protoc..

[13] van Vliet M.T.H., Jones E.R., Flörke M., Franssen W.H.P., Hanasaki N., Wada Y., Yearsley J.R. (2021). Global water scarcity including surface water quality and expansions of clean water technologies. Environ. Res. Lett..

[14] Tucker K., Stone W., Botes M., Feil E.J., Wolfaardt G.M. (2022). Wastewater Treatment Works: A Last Line of Defense for Preventing Antibiotic Resistance Entry Into the Environment. Front. Water.

[15] Mohamad Z.A., Bakon S.K., Mohamad Jamil N., Mustafa G., Thoms-Rodriguez C.-A., Mullings J., Akpaka P.E., Roye-Green K.J., McIntosh-Morgan V., Arif R. (2025). From Rivers to Tap: The Spread of Antimicrobial Resistance in Drinking Water Systems. Antimicrobial Resistance—New Insights.

[16] Heuer H., Krögerrecklenfort E., Wellington E.M., Egan S., van Elsas J.D., van Overbeek L., Collard J.M., Guillaume G., Karagouni A.D., Nikolakopoulou T.L. (2002). Gentamicin resistance genes in environmental bacteria: Prevalence and transfer. FEMS Microbiol. Ecol..

[17] Chow L.K.M., Ghaly T.M., Gillings M.R. (2021). A survey of sub-inhibitory concentrations of antibiotics in the environment. J. Environ. Sci..

[18] Ashbolt N.J., Amézquita A., Backhaus T., Borriello P., Brandt K.K., Collignon P., Coors A., Finley R., Gaze W.H., Heberer T. (2013). Human Health Risk Assessment (HHRA) for environmental development and transfer of antibiotic resistance. Environ. Health Perspect..

[19] Gullberg E., Cao S., Berg O.G., Ilbäck C., Sandegren L., Hughes D., Andersson D.I. (2011). Selection of Resistant Bacteria at Very Low Antibiotic Concentrations. PLoS Pathog..

[20] Bakon S.K., Mohamad Z.A., Mustafa G., Thoms-Rodriguez C.-A., Mullings J., Akpaka P.E., Roye-Green K.J., McIntosh-Morgan V., Arif R. (2025). Flushed and Forgotten: Antimicrobial Resistance from Wastewater Perspective. Antimicrobial Resistance—New Insights.

[21] Sinthuchai D., Boontanon S.K., Boontanon N., Polprasert C. (2016). Evaluation of removal efficiency of human antibiotics in wastewater treatment plants in Bangkok, Thailand. Water Sci. Technol..

[22] Jones E.R., Bierkens M.F.P., Wanders N., Sutanudjaja E.H., van Beek L.P.H., van Vliet M.T.H. (2022). Current wastewater treatment targets are insufficient to protect surface water quality. Commun. Earth Environ..

[23] Rizzo L., Fiorentino A., Anselmo A. (2013). Advanced treatment of urban wastewater by UV radiation: Effect on antibiotics and antibiotic-resistant E. coli strains. Chemosphere.

[24] Hoffmann M., Fischer M.A., Neumann B., Kiesewetter K., Hoffmann I., Werner G., Pfeifer Y., Lübbert C. (2023). Carbapenemase-producing Gram-negative bacteria in hospital wastewater, wastewater treatment plants and surface waters in a metropolitan area in Germany, 2020. Sci. Total Environ..

[25] Varela A.R., Ferro G., Vredenburg J., Yanık M., Vieira L., Rizzo L., Lameiras C., Manaia C.M. (2013). Vancomycin resistant enterococci: From the hospital effluent to the urban wastewater treatment plant. Sci. Total Environ..

[26] Wyres K.L., Holt K.E. (2018). Klebsiella pneumoniae as a key trafficker of drug resistance genes from environmental to clinically important bacteria. Curr. Opin. Microbiol..

[27] Verburg I., Hernández Leal L., Waar K., Rossen J.W.A., Schmitt H., García-Cobos S. (2024). Klebsiella pneumoniae species complex: From wastewater to the environment. One Health.

[28] Müller H., Sib E., Gajdiss M., Klanke U., Lenz-Plet F., Barabasch V., Albert C., Schallenberg A., Timm C., Zacharias N. (2018). Dissemination of multi-resistant Gram-negative bacteria into German wastewater and surface waters. FEMS Microbiol. Ecol..

[29] Benoit T., Cloutier M., Schop R., Lowerison M.W., Khan I.U.H. (2020). Comparative assessment of growth media and incubation conditions for enhanced recovery and isolation of Acinetobacter baumannii from aquatic matrices. J. Microbiol. Methods.

[30] Galarde-López M., Velazquez-Meza M.E., Godoy-Lozano E.E., Carrillo-Quiroz B.A., Cornejo-Juárez P., Sassoé-González A., Ponce-de-León A., Saturno-Hernández P., Alpuche-Aranda C.M. (2024). Presence and Persistence of ESKAPEE Bacteria before and after Hospital Wastewater Treatment. Microorganisms.

[31] Kong Y., Li C., Chen H., Zheng W., Sun Q., Xie X., Zhang J., Ruan Z. (2021). In vivo Emergence of Colistin Resistance in Carbapenem-Resistant Klebsiella pneumoniae Mediated by Premature Termination of the mgrB Gene Regulator. Front. Microbiol..

[32] Cannatelli A., D’Andrea M.M., Giani T., Pilato V.D., Arena F., Ambretti S., Gaibani P., Rossolini G.M. (2013). In Vivo Emergence of Colistin Resistance in Klebsiella pneumoniae Producing KPC-Type Carbapenemases Mediated by Insertional Inactivation of the PhoQ/PhoP *mgrB* Regulator. Antimicrob. Agents Chemother..

[33] M Campos J.C.d., Antunes L.C.M., Ferreira R.B.R. (2020). Global Priority Pathogens: Virulence, Antimicrobial Resistance and Prospective Treatment Options. Future Microbiol..

[34] Mutuku C., Melegh S., Kovacs K., Urban P., Virág E., Heninger R., Herczeg R., Sonnevend Á., Gyenesei A., Fekete C. (2022). Characterization of β-Lactamases and Multidrug Resistance Mechanisms in Enterobacterales from Hospital Effluents and Wastewater Treatment Plant. Antibiotics.

[35] Harris S., Morris C., Morris D., Cormican M., Cummins E. (2013). The effect of hospital effluent on antimicrobial resistant E. coli within a municipal wastewater system. Environ. Sci. Process. Impacts.

[36] Joseph N.M., Bhanupriya B., Shewade D.G., Harish B.N. (2015). Relationship between Antimicrobial Consumption and the Incidence of Antimicrobial Resistance in Escherichia coli and Klebsiella pneumoniae Isolates. J. Clin. Diagn. Res..

[37] Abdullah S., Rahman S.U., Muhammad F., Mohsin M. (2023). Association between antimicrobial consumption and resistance rate of Escherichia coli in hospital settings. J. Appl. Microbiol..

[38] Sadowy E., Luczkiewicz A. (2014). Drug-resistant and hospital-associated Enterococcus faecium from wastewater, riverine estuary and anthropogenically impacted marine catchment basin. BMC Microbiol..

[39] Rowe W.P.M., Baker-Austin C., Verner-Jeffreys D.W., Ryan J.J., Micallef C., Maskell D.J., Pearce G.P. (2017). Overexpression of antibiotic resistance genes in hospital effluents over time. J. Antimicrob. Chemother..

[40] Hsu J. (2020). How covid-19 is accelerating the threat of antimicrobial resistance. BMJ.

[41] Cantón R., Horcajada J.P., Oliver A., Garbajosa P.R., Vila J. (2013). Inappropriate use of antibiotics in hospitals: The complex relationship between antibiotic use and antimicrobial resistance. Enferm. Infecc. Microbiol. Clin..

[42] Khan F.A., Söderquist B., Jass J. (2019). Prevalence and Diversity of Antibiotic Resistance Genes in Swedish Aquatic Environments Impacted by Household and Hospital Wastewater. Front. Microbiol..

[43] Kitagawa D., Komatsu M., Nakamura A., Suzuki S., Oka M., Masuo K., Hamanaka E., Sato M., Maeda K., Nakamura F. (2021). Nosocomial infections caused by vancomycin-resistant Enterococcus in a Japanese general hospital and molecular genetic analysis. J. Infect. Chemother..

[44] Camacho-Ortiz A., Flores-Treviño S., Bocanegra-Ibarias P. (2025). Prevalence of difficult-to-treat resistance in ESKAPE pathogens in a third level hospital in Mexico. Infect. Prev. Pract..

[45] Bakon S.K., Mohamad Z.A., Jamilan M.A., Hashim H., Kuman M.Y., Shaharudin R., Ahmad N., Muhamad N.A. (2023). Prevalence of Antibiotic-Resistant Pathogenic Bacteria and Level of Antibiotic Residues in Hospital Effluents in Selangor, Malaysia: Protocol for a Cross-sectional Study. JMIR Res. Protoc..

