# Supplementary material for: City or Suburb, Resistance Flows: Wastewater-Borne ESKAPE and AMR Genes in Malaysian Hospitals
Source: Antibiotics (Basel). 2025 Oct 23;14(11):1058. doi: 10.3390/antibiotics14111058 (PMC12649672; doi:10.3390/antibiotics14111058)
Supplement: Supplementary file 1 [file antibiotics-14-01058-s001.zip › antibiotics-3768850-supplementary.pdf]

Supplementary

Table S1: Primer sequence, annealing temperature, Positive control and CT-values

No.	ARGs	Primer sequences 5' – 3'	Annealing temperatures	Positive control (Ct value)	Isolate range (Ct value)
1	*VanA*	F: GGGAAAACGACAA TTGC<br>R: GTACAATGCGGCCGTTA	60°C	*Enterococcus faecium* strain EH2 16S ribosomal RNA gene, partial sequence (18.82)	Min (18.82)<br>Max (23.88)
2	*BlaTEM*	F: TCGCCGCATACACTATTCTCAGAATGA<br>R: ACGCTCACCGGCTCCAGATTTAT	60°C	*Aeromonas caviae* strain PBSCIFE-11 16S ribosomal RNA gene, partial sequence (15.16)	Min(12.27)<br>Max(25.22)
3	*ermB*	F: GATACCGTTTACGAAATTGG<br>R: GAATCGAGACTTGAGTGTGC	56°C	*Enterococcus faecium* strain EH2 16S ribosomal RNA gene, partial sequence (19.09)	Min (13.39)<br>Max (31.79)
4	*tetA*	F: GCTACATCCTGCTTGCCTTC<br>R: CATAGATCGCCGTGAAGAGG	60°C	*Klebsiella pneumoniae* strain RGUDBI02 16S ribosomal RNA gene, partial sequence (17.55)	Min (14.11)<br>Max (30.16)
5	*Sul1*	F: CACCGGAAACATCGCTGCA<br>R: AAGTTCCGCCGCAAGGCT	60°C	*Klebsiella pneumoniae* strain RGUDBI02 16S ribosomal RNA gene, partial sequence (14.87)	Min (14.25)<br>Max (28.68)

6	*BlaNDM-1*	F: GGGCAGTCGCTTCCAACGGT<br>R: GTAGTGCTCAGTGTCGGCAT	52°C	*Klebsiella* sp. JHW3 16S ribosomal RNA gene, partial sequence (25.97)	Min (23.95)<br>Max (32.1)
